# Supplementary figures and images for: Sex, Head Size, and Male Mate Location Behavior Affect Allometric Scaling of the Eyes in Centris pallida (Hymenoptera: Apidae) Bees
Source: Integr Org Biol. 2026 Apr 24;8(1):obag016. doi: 10.1093/iob/obag016 (PMC13181258; doi:10.1093/iob/obag016)

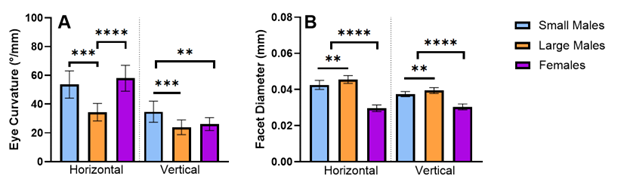

Supplement: obag016_Supplemental_Files [file obag016_supplemental_files.zip › Figure S5.tif]

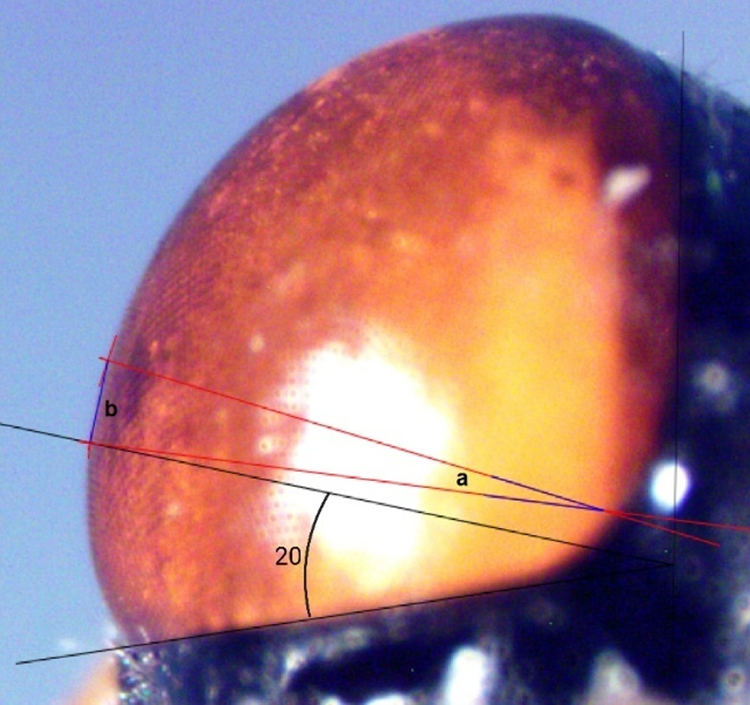

Supplement: obag016_Supplemental_Files [file obag016_supplemental_files.zip › FigureS1.tif]

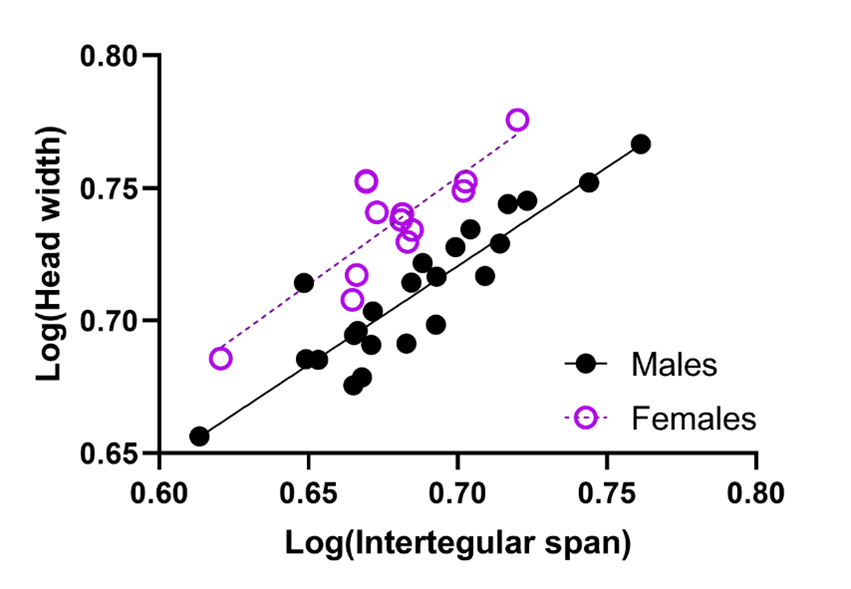

Supplement: obag016_Supplemental_Files [file obag016_supplemental_files.zip › FigureS2.tif]

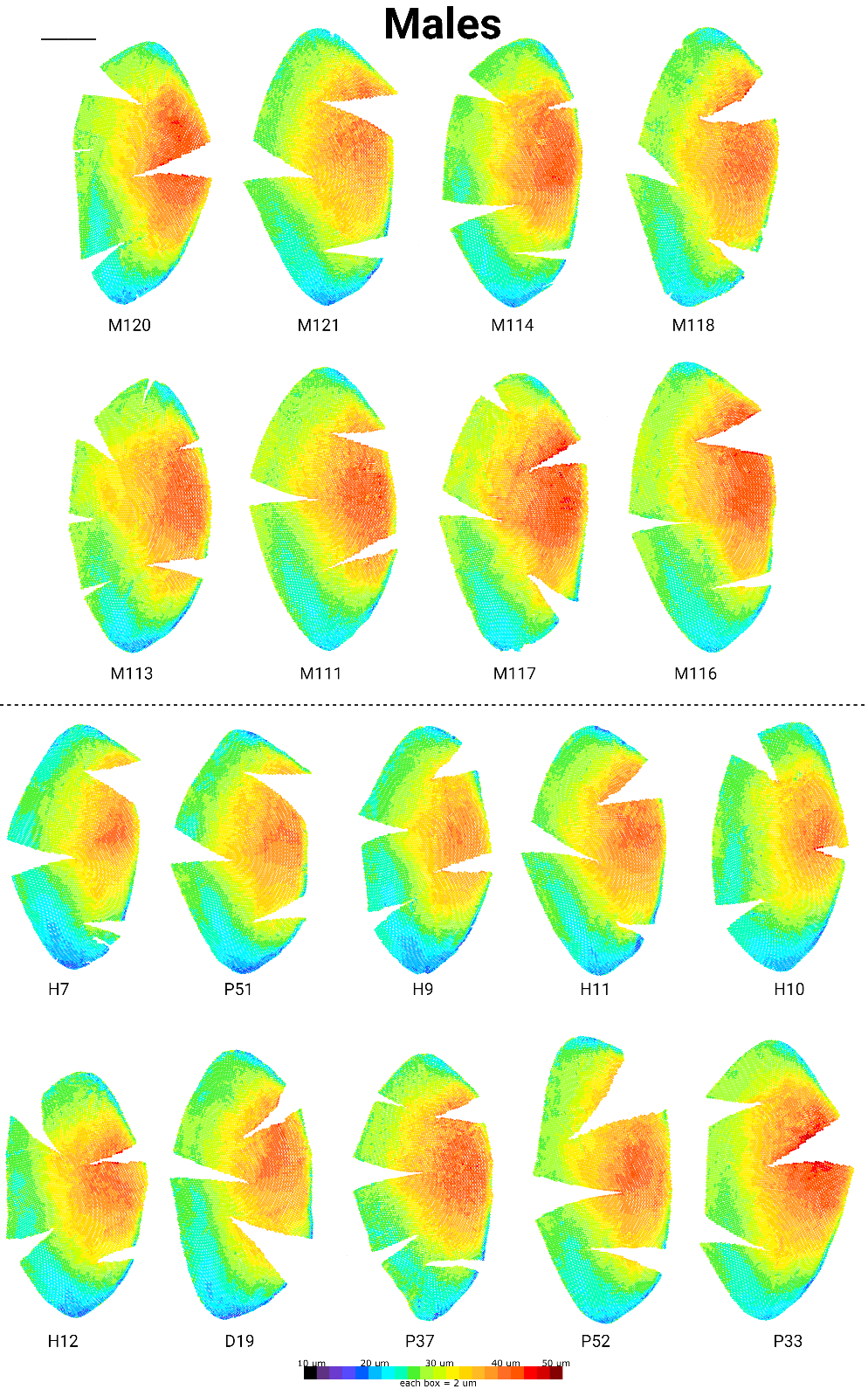

Supplement: obag016_Supplemental_Files [file obag016_supplemental_files.zip › FigureS3.tif.png]

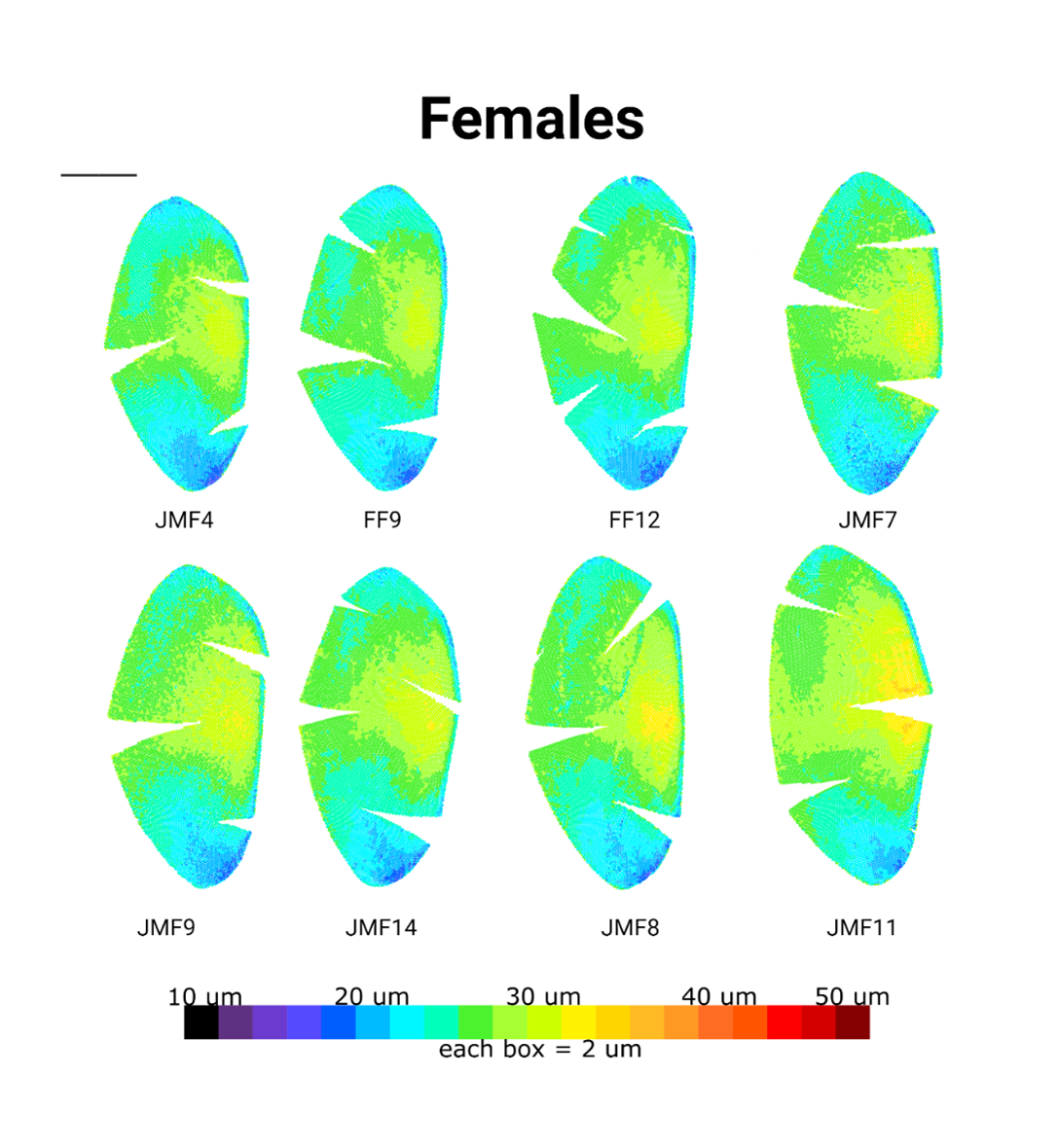

Supplement: obag016_Supplemental_Files [file obag016_supplemental_files.zip › FigureS4.tif]
